# Knowledge, Practice, and Awareness of Oral Cancer and HPV Infection among Dental Students and Residents: A Cross-Sectional Study

**DOI:** 10.3390/medicina58060806

**Published:** 2022-06-15

**Authors:** Alice Murariu, Elena-Raluca Baciu, Livia Bobu, Diana Diaconu-Popa, Irina Zetu, Gabriela Gelețu, Roxana-Ionela Vasluianu, Loredana Hurjui

**Affiliations:** 1 Department of Surgicals, Faculty of Dental Medicine, University of Medicine and Pharmacy, “Grigore T. Popa”, 700115 Iasi, Romania; alice.murariu@umfiasi.ro (A.M.); irina.zetu@umfiasi.ro (I.Z.); gabriela.geletu@umfiasi.ro (G.G.); 2 Department of Implantology, Removable Prostheses, Dental Technology, Faculty of Dental Medicine, University of Medicine and Pharmacy, “Grigore T. Popa”, 700115 Iasi, Romania; diana.diaconu@umfiasi.ro (D.D.-P.); roxana.vasluianu@umfiasi.ro (R.-I.V.); 3 Department of Morpho-Functional Sciences II, Faculty of Medicine, University of Medicine and Pharmacy, “Grigore T. Popa”, 700115 Iasi, Romania; loredana.hurjui@umfiasi.ro

**Keywords:** oral cancer, HPV, knowledge, practice, dental students, residents

## Abstract

*Background and Objectives:* Dentists play a very important part in the early identification of oral cancer lesions. This aspect of dental practice depends on the knowledge acquired during the faculty years. The aim of this study was to assess dental students’ and residents’ levels of awareness in terms of oral cancer. *Materials and Methods:* The cross-sectional study was conducted at the Faculty of Dental Medicine within the “Grigore T. Popa” University of Medicine and Pharmacy in Iasi, on a sample of 197 students in the fourth and the fifth years and first year residents in general dentistry. To assess their knowledge, a questionnaire was created containing 22 questions about the risk factors for oral cancer, with a focus on HPV infection. *Results:* Most participants correctly identified smoking, alcohol, and the HPV infection as risk factors and leukoplakia and erythroplasia as potentially malignant lesions. At the opposite site, aspects considered as unsatisfactory focused on the palpation of lymphatic nodules, a procedure largely carried out by 41.6% of the fourth year students, the counseling only of the patients at risk performed by 59.7% of residents, the lack of knowledge about the prevention of oral cancer through anti-HPV immunization found in 39.7% of the fifth year students. Other incorrect answers focused on other types of suspicious lesions, such as actinic cheilitis, as well as certain areas in the oral cavity subject to the frequent onset of oral cancer, such as buccal mucosa. *Conclusions:* Although the fifth year students and residents have better knowledge than the fourth year students, the gaps in terms of knowledge and practice encountered in all three categories of participants require a reevaluation of the academic curriculum and the focus on the building of the skills necessary for the correct screening of oral cancer.

## 1. Introduction

According to World Health Organization (WHO) [1], cancer represents the second cause of death in the world, with a number of 10 million deaths in 2020. In low-income countries, almost 30% of cases are attributed to infections such as hepatitis or the infection caused by the Human Papilloma Virus (HPV). In Europe, the incidence of cancer at the age of 0–74 in men is 30.74%, while in women it is 22.18% [2].

Oral cancer is a major public health issue due to the high cost of treatment, permanent disability, and the depreciation of life quality [3]. Oral cancer is the most common form of cancer at head-throat level, and the form of Squamous cell carcinoma is the most frequent among all malignant forms [3]. Oral and oropharyngeal cancer include cancer of the tongue, lip, floor of the mouth, gingiva, palate and buccal mucosa, alveolar mucosa, and oropharynx, as well as the pharyngeal tonsils and salivary glands. Males are twice as likely as females to be diagnosed with oral and oropharyngeal cancer [4].

According to the data provided by the International Agency for Research in Cancer (GLOBOCAN 2020) [2], the number of new cases of oral cancer in 2020 in the world was 377,713, while the number of deaths was 177,757.

Approximately 450,000 new lip and oral cavity cancer cases are diagnosed each year, with only 40–50% of patients surviving for the next 5 years after the diagnosis [5,6].

The prevalence of oral cancer is higher in Asian countries, such as India, Sri Lanka, Pakistan, Bangladesh, and Melanesia, following one common habit in these countries, namely betel quid chewing [7]. Eastern Europe is one of the areas with the highest age standardized incidence rate of oral cancer worldwide [8]. The highest values in men correspond to Hungary (18.7), Slovakia (14.0), and Romania (13.6); in women, the highest rates are observed in Hungary (3.3), Slovakia (1.3), and Poland (1.2) [9].

In Romania, 1929 new cases of oral cancer were registered in 2020, and there were 901 deaths with a 5-year survival rate of 28.05 for 100,000 individuals [10].

Although oral cancer is a disease that can be prevented, the main risk factors, smoking and alcohol consumption, are present in 90% of the cases, their action being synergic [11].

Among other risk factors, there is human papillomavirus (HPV), associated with oral-pharyngeal carcinoma, Epstein-Barr virus, and ultraviolet radiation, frequently causing lip cancer [12,13]. HPV includes a family of DNA viruses that infect basal epithelial cells, causing benign and malignant lesions of the skin and mucosae of the anogenital and upper aero-digestive tract [14,15].

Official data of WHO confirm that types 16 and 18 of more than 200 types of Human Papilloma Virus are most frequently incriminated in the onset of oral-pharyngeal cancer [16,17]. Most HPV infections are asymptomatic and tend to spontaneously heal within 2 weeks, but there is the possibility of a persistent infection that will result in genital cancer and oral-pharyngeal cancer, with a higher frequency in men [18].

HPV vaccination may also be useful for preventing HPV-related cancers other than cervical cancer. For example, most cases of oropharyngeal carcinoma are caused by HPV 16 (about 90%) and HPV 18, and HPV vaccination for this condition can be expected to have a greater disease-suppressing effect than in cervical cancer [19].

The immunization of the population at the age of 12, before people become sexually active, is essential in preventing these types of cancers.

Dentists play an essential role in the primary prevention of oral cancer by informing their patients about the importance of avoiding the major risk factors and making regular dental visits [20,21].

A simple correct and full examination of the areas prone to develop oral cancer can make the difference between life and death. This means that dentists should have the proper knowledge about the characteristics of lesions and their location, as well as the management of the patient with suspicious lesions. Literature shows that some physicians and dentists possess insufficient knowledge regarding oral cancer, though courses exist on this topic in the curriculum of the relevant faculties [22,23,24,25,26].

As for the curriculum of the Faculty of Dental Medicine of Iasi, only general information about cancer is provided to students in their first years of study, while the student’s direct contact with these theoretical and practical notions takes place only in the fifth year of study in Oral and maxillofacial Surgery (OMF Surgery) in the first/second semester.

The preparation for general dentistry residency also contains a 3-month OMF Surgery module in the third year of training.

The aim of this study was to assess the knowledge of the fourth and fifth year students attending the Faculty of Dental Medicine within the “Grigore T. Popa” University of Medicine and Pharmacy of Iasi and the knowledge of the first year residents in general dentistry in relation to oral cancer, namely risk factors, clinical aspect, anatomic locations, types of neoplastic lesions, practices, and prevention. Moreover, the research was expanded to verify their knowledge and attitude in terms of the Human Papilloma Virus infection, namely transmission, symptomatology, immunization, and prevention.

The null hypothesis tested in the study was that there was no difference in knowledge and practice between fourth and fifth year students and residents of general dentistry regarding oral cancer and HPV infection.

## 2. Materials and Methods

### 2.1. Research Design

The cross-sectional study was conducted in February–March 2022 at the Faculty of Dental Medicine of Iasi on a sample of 197 students in the fourth and fifth years and first year residents in general dentistry.

To conduct this study, the approval (No. 156/23 February 2021) of the ethics committee of “Grigore T. Popa” University of Medicine and Pharmacy of Iasi was obtained, as well as the informed consent of the participants to the study. Their anonymity was ensured, and they were offered the possibility to refuse participation to the study.

### 2.2. The Study Group

A convenience sampling method was used in this study. Initially, 250 eligible students and residents were invited to fill in a questionnaire specially designed for this research. A number of 37 of them failed to answer the invitation, and 16 questionnaires were rejected as being incomplete; therefore, the final number of students and residents included in this study that fully completed the questionnaire was 197, providing a participation rate of 78.8%.

### 2.3. The Study Instrument

The questionnaire used in this study was designed after an extended review of the literature [16,21,24,27,28,29], and it was adequate for the study’s purpose, namely the assessment of knowledge and attitude towards the practice and primary prevention of oral cancer, in general, and the HPV infection, in particular.

The original questionnaire was translated into Romanian by two expert linguists using forward and back-translation. The final version of the self-administered questionnaire included 22 questions and it was tested for internal consistency and validated.

Before conducting the main study, a pilot study was conducted on a group of 15 students in order to verify the level of comprehension and coherence of the questions. Finally, minor adjustments were necessary so that the questionnaire should be easy to understand and fill in (5 min). The questionnaire contained 4 sections.

The first section contained social-demographic information, such as age, gender, and year of study.

The second section contained 4 questions regarding the vulnerable age group (Q1), risk factors for oral cancer (Q2), areas in the oral cavity prone to the onset of oral cancer (Q3), and types of pre-malignant lesions (Q4).

The third section had 9 items and assessed students’ and residents’ knowledge in terms of examination of oral mucosa (Q5), palpation of lymphatic nodules (Q6), and characteristics of suspicious lesions (Q7). The following 3 questions referred to patients’ counseling in terms of the risk factors for oral cancer (Q8), the correctness of the clinical examination performed by dentists (Q9), and the potential obstacles against a correct examination, such as the lack of necessary time, skills, and expertise, and financial incentives (Q10).

The last three questions of this section referred to the perception towards continuous medical training (Q11), the desire to receive more information (Q12), and the types of continuous medical training preferred by the participants (Q13).

The fourth section contained 9 questions relating to the knowledge on HPV infection: the correlation between HPV and cervical cancer, oral-pharyngeal cancer, and AIDS disease (Q14, Q15, Q16), the method of transmission (Q17), the clinical picture (Q18), the anti-HPV immunization (Q19), the prevention of genital and oral cancer (Q20, Q21), and the adequate age for immunization (Q22).

### 2.4. Data Analysis

Statistical analysis was performed using the Statistical Package for Social Sciences program (SPSS Inc., Chicago, IL, SUA, version 21 for Windows). Descriptive data were analyzed using frequency, percentage, and crosstabulation, and the Chi-square test was used to identify differences between groups, with a significance level of 5% (*p* < 0.05).

## 3. Results

### 3.1. Study Participants

Out of the 197 participants, 77 (39.1%) were fourth year students, 63 (32%) were fifth year students, and 57 (28.9%) were first year residents in general dentistry. The age range of study participants was 21–43 years, with a mean of 25.01 ± 4.850.

The gender distribution in the study sample was 71 males (36%) and 126 females (64%), following the gender distribution of the students in the faculty.

The characteristics of the study participants are presented in Table 1.

### 3.2. Knowledge of the Clinical Practice and Risk Factors for Oral Cancer

**Figure 1:**
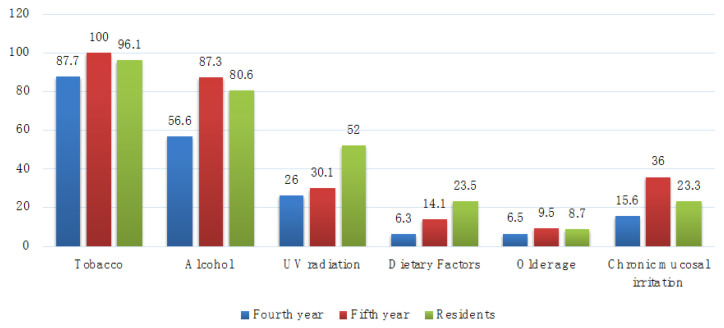
Percentage of participants that correctly identified the risk factors for oral cancer (Q2).

Among the risk factors for oral cancer (Figure 1), smoking and alcohol were correctly identified by at least 87.7% and 56.6% of participants, respectively. Chronic mucosal irritation was identified by most of the fifth year students (36%), dietary factors were identified by 23.5% of the residents, and older age was identified by 9.5% of the fifth year students.

**Figure 2:**
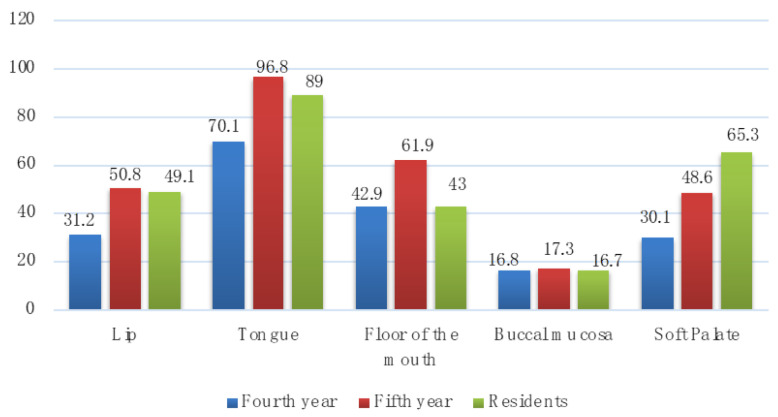
Percentage of participants that correctly identified most common sites for oral cancer (Q3).

Question Q3 assessed the knowledge on the oral cavity areas prone to oral cancer (Figure 2). Most participants considered the tongue as a risky area for oral cancer: 96.8% of the fifth year students, 89% of residents, and 70.1% of the fourth year students. The soft palate was identified as a risky area by 65.3% of residents, 48.6% of the fifth year students, and 30.1% of the fourth year students. The floor of the mouth was identified by 61.9% of the fifth year students, the lip was identified by 50.8% of the fifth year students, and buccal mucosa had a percentage of correct answers below 18%.

The answers provided by participants to questions Q1 and Q4–Q13 are presented in Table 2. The correct answer to Q1 (>40 years) was provided by the vast majority of the participants: 84.4% of fourth year students, 74.6% of fifth year students and 89.5% of residents (*p* = 0.005). The most well-known neoplastic lesion (Q4) was leukoplakia, in the highest percentage by the fifth year students (77.7%), followed by residents (71.9%) and fourth year students (71.4%). The least identified lesion was actinic cheilitis, in the highest percentage by the fifth year students (23.8%), followed by the residents (8.7%) and the fourth year students (2.6%).

All fifth year students stated that they usually examine the oral mucosa (Q5), followed by 98.2% of residents and 85.7% of the fourth year students. Lymphatic nodules palpation (Q6) was performed on a regular basis by 41.6% of the fourth year students, 36.5% of the fifth year students, and 24.6% of residents. Indurated nodules as a characteristic of neoplastic lesions (Q7) were identified in the highest percentage by the fifth year students (58.7%), while 22% of the fourth year students could not provide the correct answer. In terms of counseling the patient on the primary prevention of oral cancer (Q8), most of the fourth and fifth year students stated that they did it for every patient (53.2% and 77.9%, respectively), while most of residents (59.7%) said they counseled only risk patients.

Most of the respondents considered that dentists do not always make a correct clinical examination to identify the potential suspicious lesions (Q9), with the highest percentage in the residents’ group (96.5%). The most probable causes for this failure to perform a correct clinical examination (Q10) were considered to be lack of time (31.2% of the fourth year students and 39.7% of the fifth year students) and lack of experience (52.6% of the residents). The majority of participants considered that they did not have sufficient knowledge about the forms of oral cancer (Q11), with the highest percentage in the fourth year students’ group (92.2%). A percentage of more than 96% of the respondents expressed their desire to receive further information (Q12), with most of them preferring practical training (Q13) (57.1% in the fourth year students’ group and 65.0% in the fifth year students’ group) or information distributed in the faculty (35.0% in residents’ group).

Significant differences were found between the three groups for questions Q1 (*p* = 0.048), Q4 (*p* = 0.001), Q5 (*p* = 0.001), Q7 (*p* = 0.003), Q8 (*p* = 0.000), Q10 (*p* = 0.000), Q11 (*p* = 0.000), and Q13 (*p* = 0.000).

### 3.3. Knowledge about HPV Risk Factor for Oral Cancer

The final nine questions of the questionnaire assessed knowledge in terms of HPV infection (Table 3).

HPV was identified as the etiologic agent of cervical cancer (Q14) by most of the fifth year students and residents (63.5% and 63.2%, respectively) and oral cancer (Q15) by most residents (70.2%) (*p* = 0.002). HPV involvement in AIDS etiology (Q16) was correctly denied by most of the residents (61.4%), with the highest percentage of the three groups (*p* = 0.000).

The sexual and oral ways of HPV transmission (Q17) were correctly identified both by the fifth year students and residents, (49.2% and 47.4%, respectively), but not by the fourth year students (37.7%) (*p* = 0.014).

The fact that HPV infection is asymptomatic (Q18) was known by 80.7% of residents, 69.8% of the fifth year students, and 59.7% of the fourth year students, with significant differences between groups (*p* = 0.011).

Most participants were aware of the existence of an anti-HPV vaccine (Q19) and the possibility of preventing cervical cancer by vaccination (Q20), but not about the possibility of preventing oral cancer by vaccination (Q21). However, significant differences were found for the three questions (*p* = 0.006, *p* = 0.003, and *p* = 0.003, respectively).

The recommended age for anti-HPV immunization (Q22) was known by most of the subjects, with higher percentages among the fifth year students and residents (68.3% and 68.4%, respectively) than the fourth year students (39.0%) (*p* = 0.000).

## 4. Discussion

The early detection of oral cancer has important benefits for the patient in terms of diagnostic, surgical treatment, which is less invasive and, of course, preserves the quality of life [30].

That is why the acquisition of some practical skills for the recognition of suspicious malignant lesions in the oral cavity must necessarily be done during faculty. Although in the first 4 years students study Microbiology and Morphopathology, comprising some general elements of oral cancer, it is only in the fifth year, after having completed the OMF Surgery training courses, that the students have information about the etiology, the clinical picture, and the types of suspicious lesions. In this context, it is obvious that the fourth year students have serious gaps as they are incapable of identifying some types of malignant lesions, such as actinic cheilitis, identified by 2.6% students.

However, exceptions were encountered, such as the transmission of HPV, where the correct answers were recorded in a higher percentage among the fourth year students (44.2%), compared to the fifth year students (36.5%) or residents (21.1%). This can be explained by the fact that, although the information provided in the OMF Surgery course is sufficiently detailed, it is still not exhaustive. Such gaps may indicate the need for a subsequent modification of the curriculum.

The highest number of correct answers concerning potentially malignant lesions was provided by the fifth year students; the most known lesion was leukoplakia (77.7%), followed by erythroplasia (60.3%) and lichen planus (44.4%).

Similar results were obtained by Galina et al. [21] in Brasil, where dental students identified actinic cheilitis in a percentage of 2%, while erythroplasia was identified by 32% of students.

The present study highlighted the lack of knowledge about some of the risk factors for oral cancer, such as dietary factors recognized in the highest percentage by residents and older age and chronic mucosal irritation identified by most fifth year students. In exchange, smoking and alcohol consumption were correctly identified by most participants of all three categories, possibly due to the information received during pre-clinical training and from other supplementary sources, such as the academic educational programs and those mentioned in the medical journals.

These results are similar to those from studies conducted in Jordan [16], Croatia [22], Spain [31], and Nepal [32]. Contrary to these results, Tadbir [33] in Iran found a lower percentage of students who correctly identified smoking (40.7%) and alcohol (47.4%) as risk factors.

The highest number of correct answers concerning the potential localizations of oral cancer was given by the fifth year students, who identified the tongue (96.8%), the floor of the mouth (61.9%), the lip (50.8%), and buccal mucosa (17.3%). The soft palate was correctly identified by most residents, at 65.3%.

In a study conducted in 2014 in Romania, at the University of Bucharest by Dumitrescu et al. [34], 96.8% and 77.7% of dental students identified smoking and alcohol consumption, respectively, as risk factors for oral cancer, while 87.7% and 54.3% of them knew that the tongue and the floor of the mouth, respectively, are common locations for oral cancer.

The present study showed that the correct clinical examination is only partially performed by dental students and residents. Although more than 85.7% of them examine the oral mucosa, the palpation of lymphatic nodules is performed by only a low percentage, with the highest proportion among the fourth year students (41.6%). One of the reasons could be that this maneuver might not be presented well enough during the training stages, which means that it is necessary to identify the drawbacks of the academic curriculum so that future professionals may have the necessary practical skills.

Patients’ counseling in terms of the risk factors for oral cancer and the symptoms of suspicious lesions are essential elements that must be applied to all patients, not only to those at risk (as 59% of residents answered). This is a fairly common attitude among dentists, to consider that the patient who smokes and drinks alcohol would not change this behavior; therefore, education in this would not be successful [35]. In this sense, in the present study, among the possible barriers in achieving a correct clinical practice, first, the lack of time and the lack of experience were mentioned.

There were questions in this study as to whether the percentage of correct answers was higher in fifth year students than in residents, such as those referring to: the risk factor-chronic mucosal irritation (36% vs. 23.3%); common sites for oral cancer—floor of the mouth (61.9% vs. 43%); lymph node palpation (77.9% vs. 10.5%); and vaccine against HPV (82.5% vs. 78.9%). One possible explanation would be that some of the residents have a higher number of years since graduation and have not faced such cases in their current practice during this time.

The need for additional information was recognized by more than 96% of participants in the present study, and among the forms of continuous medical training, the practical form was mentioned by most students (65.1% of the fifth year students and 57.1% of the fourth year students). In other similar studies, students and dentists asked for further information of workshop types [16,29,36] or the attendance of supplementary training courses [37].

HPV involvement in oral and oropharyngeal carcinogenesis was first proposed by Syrjanen et al. in 1983. In 2007, the International Agency for Research on Cancer recognized human papillomavirus type 16 (HPV16) as the only carcinogenic type of HPV in sites other than the cervix uteri, including oral cavity and oropharynx [38].

Dentists and dental students play an important role in the prevention of HPV infection if they counsel their patients in terms of the methods of transmission, clinical picture, and immunization. In the present study, only 17.5% of the residents identified oral intercourse to be a risk factor for oral cancer, as compared to 50.8% of the students of the University of Benin [39].

The results of the present study showed that 70.2% of residents, 42.9% of the fourth year students, and 34.9% of the fifth year students identified the HPV infection as an etiological factor for oral cancer. Similar results were also obtained in other studies. Poelman et al. [28], in the Netherlands, found a percentage of 75% of master’s degree students and 54.3% of dental students, and Sallam [16] found even higher percentages among dental students in Jordan (97.2%).

Correct answers were also obtained for other questions, such as those referring to: HPV as an etiological agent of cervical cancer (63.2% of residents); HPV infection being asymptomatic (80.7% of residents); the existence of a vaccine against HPV (82.5% of the fifth year students), and the age when the anti-HPV vaccine is taken (68.4% of residents).

There were also questions that many of the participants did not know how to answer, such as those referring to: the involvement of HPV infection in AIDS etiology (54% of the fifth year students and 36.8% of residents); the possibility of preventing cervical cancer by vaccination (38.6% of residents and 22.1% of the fourth year students), and the possibility of preventing oral cancer by vaccination (61.4% of residents). Contrary to these results, Rajiah [40] in Malaysia found a higher percentage of students (68.9%) who knew that the vaccine might prevent cervical cancer.

As for the vaccination age, a percentage of 35.0% of the fourth year students and 28.1% of residents did not know the answer. A possible explanation might be that the academic curriculum does not contain any information about the HPV infection, mainly about the benefits of the anti-HPV vaccination. These results coincide with those of similar studies, which also showed that students of more advanced years of study and the residents had higher knowledge of oral cancer. Additionally, they had a more developed awareness of risk factors, and it is a well-known fact that awareness regarding risk factors is a prerequisite for the prevention of oral cancer [23].

The results of the study demonstrate the existence of statistically significant differences in the knowledge of the three categories of subjects, namely, fourth year students, fifth year students, and residents, which means that the null hypothesis is rejected.

This study has a series of limitations: first of all, the predominance of female respondents in the groups of participants who may have influenced some answers, especially to the questions relating to HPV infection, methods of transmission, and vaccination. The second limitation refers to the fact that the results do not reflect the knowledge of all students and residents of the faculty or from other Romanian universities.

A follow-up comparative study is recommended among medical students and medical residents or other professionals, such as general practitioners.

## 5. Conclusions

The following conclusions were formulated based on the study’s findings:

- In general, most fifth year students and residents have more solid knowledge about oral cancer than fourth year students;
- Satisfactory knowledge was noticed in terms of the risk factors and areas in the oral cavity prone to cancer;
- Leukoplakia was the type of pre-malignant lesion identified by most participants;
- A limited number of participants perform lymphatic ganglions palpation;
- Participants’ knowledge in terms of HPV infection may be considered as adequate, with the highest number of correct answers among the fifth year students, followed by residents and the fourth year students.

The study highlights the need for adaptation of the academic curriculum and continuous post-academic medical training, mainly practical, with a focus on building the necessary skills for the correct screening of oral cancer.

## Figures and Tables

**Table 1:** Characteristics of study participants (N = 197).

**Variables**		**N**	**%**
	**Gender**		
	Male	71	36.0
	Female	126	64.0
	**Age (mean ± SD ^1^)**	25.01 ± 4.850	
	22–24 years	135	68.5
	25–29 years	37	18.7
	>30 years	25	12.8
	**Participants**		
	4th year	77	39.1
	5th year	63	32.0
	Residents	57	28.9

^1^ SD-standard deviation.

**Table 2:** Assessment of oral cancer practice and continuing education among participants.

	4th Year	5th Year	Residents	*p*-Value
	N (%)	N (%)	N (%)	
Q1. Which is the most vulnerable age group for oral cancer?				
<18 years	2 (2.6)	3 (4.8)	0	0.048 *
19–39 years	10 (13)	13 (20.6)	6 (10.5)	
>40 years	65 (84.4)	47 (74.6)	51 (89.5)	
<18 years	2 (2.6)	3 (4.8)	0	
Q4. Identify at least one neoplastic lesion				
Erythroplasia	34 (44.2)	38 (60.3)	25 (43.9)	0.001 *
Leukoplakia	55 (71.4)	49 (77.7)	41 (71.9)	
Lichen Planus	24 (31.1)	28 (44.4)	21 (36.8)	
Actinic cheilitis	2 (2.6)	15 (23.8)	5 (8.7)	
Q5. Do you usually examine the oral mucosa?				
Yes	66 (85.7)	63 (100)	56 (98.2)	0.001 *
No	11 (14.3)	0	1 (1.8)	
Q6. Do you usually perform lymph node palpation?				
Yes	32 (41.6)	23 (36.5)	14 (24.6)	0.170
No	45 (58.4)	40 (63.5)	43 (75.6)	
Q7. What are the characteristics of oral lesions that may become malignant?				
Ulcers	13 (16.9)	20 (31.7)	20 (35.1)	0.003 *
Indurated nodules	43 (55.9)	37 (58.7)	30 (52.6)	
Abscess	4 (5.2)	4 (6.4)	5 (8.8)	
I do not know	17 (22)	2 (3.2)	2 (3.5)	
Q8. How often do you counsel your patients in terms of primary prevention of oral cancer?				
Every patient	41 (53.2)	49 (77.9)	6 (10.5)	0.000 *
Occasionally	11 (14.3)	2 (3.3)	17 (29.8)	
Only patients at risk	25 (32.5)	12 (19.0)	34 (59.7)	
Q9. Do you think that dentists always make a correct clinical examination to identify the potential suspicious lesions?				
Yes	10 (13.0)	4 (6.3)	2 (3.5)	0.115
No	67 (87.0)	59 (93.7)	55 (96.5)	
Q10. What are the causes for the clinical examination not performed correctly?				
Lack of time	24 (31.2)	25 (39.7)	16 (28.0)	0.000 *
Lack of knowledge	4 (5.2)	3 (4.8)	1 (1.8)	
Lack of abilities	3 (3.8)	3 (4.8)	7 (12.3)	
Lack of experience	16 (20.8)	17 (27)	30 (52.6)	
Lack of financial incentives	21 (27.3)	11 (17.5)	1 (1.8)	
I do not know	9 (11.7)	4 (6.3)	2 (3.5)	
Q11. Do you consider that you have sufficient knowledge about the forms of oral cancer?				
Yes	6 (7.8)	5 (23.8)	23 (40.4)	0.000 *
No	71 (92.2)	48 (76.2)	34 (59.6)	
Q12. Would you like to get more information?				
Yes	75 (97.4)	61 (96.8)	56 (98.2)	0.423
No	2 (2.6)	2 (3.2)	1 (1.8)	
Q13. What type of education do you prefer?				
Information distributed in the faculty	2 (2.6)	2 (3.2)	20 (35.0)	0.000 *
Additional courses	2 (2.6)	5 (7.9)	0 (0.0)	
Practical training	44 (57.1)	41 (65.0)	21 (36.9)	
All	29 (37.7)	15 (23.9)	16 (28.1)	

Note: Chi square test. * Significance level of 0.05.

**Table 3:** Assessment of HPV knowledge among study participants.

	4th Year	5th Year	Residents	*p*-Value
	N (%)	N (%)	N (%)	
Q14. Is the HPV an etiological agent of cervical cancer?				
Yes	43 (55.8)	40 (63.5)	36 (63.2)	0.477
No	15 (19.5)	12 (19.0)	6 (10.5)	
I do not know	19 (24.7)	11 (17.5)	15 (26.3)	
Q15. Is the HPV an etiological agent of oral cancer?				
Yes	22 (28.5)	23 (36.5)	40 (70.2)	0.002 *
No	33 (43.0)	22 (34.9)	9 (15.8)	
I do not know	22 (28.5)	18 (28.6)	8 (14.0)	
Q16. May this infection cause AIDS?				
Yes	17 (22.1)	13 (20.6)	1 (1.8)	0.000 *
No	26 (33.8)	16 (25.4)	35 (61.4)	
I do not know	34 (44.2)	34 (54.0)	21 (36.8)	
Q17. What is the method of transmission of HPV?				
Sexual	34 (44.2)	23 (36.5)	12 (21.1)	0.014 *
Oral	9 (11.6)	9 (14.3)	10 (17.5)	
Sexual and Oral	29 (37.7)	31 (49.2)	27 (47.4)	
I do not know	5 (6.5)	0 (0.0)	8 (14.0)	
Q18. Is HPV infection asymptomatic?				
Yes	46 (59.7)	64 (69.8)	46 (80.7)	0.011 *
No	9 (11.7)	12 (19.0)	5 (8.8)	
I do not know	22 (28.6)	7 (11.1)	6 (10.5)	
Q19. Is there any vaccine against HPV?				
Yes	63 (81.8)	52 (82.5)	45 (78.9)	0.006 *
No	4 (5.2)	8 (12.7)	0 (0.0)	
I do not know	10 (13.0)	3 (4.8)	12 (21.1)	
Q20. Do you think that vaccination may prevent cervical cancer?				
Yes	50 (64.9)	44 (69.8)	31 (54.4)	0.003 *
No	10 (13.0)	13 (20.6)	4 (7.0)	
I do not know	17 (22.1)	6 (9.6)	22 (38.6)	
Q21. Do you think that vaccination may prevent oral cancer?				
Yes	18 (23.4)	22 (34.9)	11 (19.3)	0.003 *
No	22 (38.6)	25 (39.7)	11 (19.3)	
I do not know	37 (48.1)	16 (25.4)	35 (61.4)	
Q22. What is the recommended age for HPV vaccine?				
11–12 years	30 (39.0)	43 (68.3)	39 (68.4)	0.000 *
>18 years	20 (26.0)	9 (14.3)	2 (3.5)	
I do not know	27 (35.0)	11 (17.4)	16 (28.1)	

Note: Chi square test. * Significance level of 0.05.

## Data Availability

The data that support the findings of this study are available on request from the corresponding author.
